# Cardiomyopathy as indication for pediatric heart transplantation

**DOI:** 10.1016/j.jhlto.2025.100360

**Published:** 2025-08-07

**Authors:** Alireza Raissadati, Drishti Tolani, Elizabeth L. Profita

**Affiliations:** a Department of Pediatrics (Cardiology), Stanford University, Palo Alto, California; b Department of Pediatrics (Cardiology), Cleveland Clinic, Cleveland, Ohio

**Keywords:** pediatric, heart transplant, dilated cardiomyopathy, hypertrophic cardiomyopathy, restrictive cardiomyopathy, heart failure

## Abstract

Pediatric cardiomyopathy (CMP), with an incidence of approximately 1 in 100,000 children, remains a critical clinical challenge. Often progressive, these myocardial disorders frequently culminate in end-stage heart failure, establishing CMP as the primary indication for pediatric heart transplantation. Key considerations for transplantation include specific CMP etiologies and subtypes alongside established listing criteria and strategies to navigate waitlist complexities. The evolving role of mechanical circulatory support, especially ventricular assist devices (VADs), has transformed bridging strategies and significantly improved survival to transplant. Contemporary post-transplant outcomes demonstrate continued improvement, with advancements in survival rates and ongoing refinement of essential long-term management strategies. This review provides a comprehensive analysis of pediatric CMP as it progresses to end-stage heart failure requiring transplantation. It synthesizes current knowledge on etiologies, clinical presentations, transplant evaluation, the transformative impact of VADs, and contemporary post-transplant management, aiming to equip clinicians with an updated framework for complex decision-making and optimizing outcomes in this high-risk population.

## Background

Pediatric cardiomyopathy (CMP) has an incidence of 1 in 100,000 children.^1^ It presents as a heterogeneous group of myocardial disorders with wide-ranging clinical phenotypes. Nearly 40% of children with severe presentations die or need transplant within 2 years of diagnosis.^2^ CMP is the indication for over half of all pediatric heart transplants.^1^ Many cases have a genetic basis, with estimates suggesting hereditary etiologies in at least 40%.^3^ Dilated cardiomyopathy (DCM) is the leading indication for heart transplantation within this population.^4^ Heart transplant is considered when medical management is insufficient to manage heart failure symptoms.

The most common type of CMP is DCM, followed by hypertrophic cardiomyopathy (HCM). Rare types include restrictive cardiomyopathy (RCM), left-ventricular noncompaction cardiomyopathy (LVNC), and arrhythmogenic cardiomyopathy (AC). Distinctions among pediatric CMP subtypes are critical for prognosis and management. Mechanical bridging strategies with ventricular assist devices (VADs) have altered the waitlist landscape, increasing survival until transplant. Post-transplant survival for children continues to improve, with median survival of 24 years for infant transplants and 14 years for adolescents, with outcomes tending to be superior in patients with DCM as compared to those with congenital heart disease (CHD).^5^ This review outlines the main pediatric CMP subtypes, highlights considerations for transplant listing, and summarizes outcomes with contemporary management.

## Types of cardiomyopathy

The initial evaluation of pediatric CMP relies on a combination of detailed patient history, physical examination, electrocardiography, and echocardiography. These tools form the backbone of the diagnostic workup. Beyond these initial steps, advanced modalities are crucial for defining the specific CMP subtype and underlying etiology. Cardiac magnetic resonance (CMR) imaging provides superior anatomical detail and tissue characterization.^6^ Comprehensive genetic, metabolic, and infectious investigations are also paramount for identifying potentially reversible causes and guiding therapies.^7^ Furthermore, genetic testing helps identify familial phenotypes, facilitates essential cascade screening within families, informs family planning, helps determine recurrence risk,^8^ and can identify potential peri-transplant risk factors related to underlying metabolic or immunologic dysfunction.^9^

### Dilated cardiomyopathy (DCM)

DCM is the most common form of CMP and is defined by left ventricular dilation and systolic dysfunction, occurring in the absence of structural heart disease or abnormal loading conditions such as hypertension or valvular disease.^4^, ^10^, ^11^ DCM etiologies can be broadly categorized as primary or secondary.

Primary DCM includes cases previously termed idiopathic, which historically accounted for 50–70% of pediatric cardiomyopathies; however, this proportion is decreasing as genetic diagnostic capabilities improve.^2^, ^4^ A genetic diagnosis is found in 30–50% of pediatric cases, most inherited in an autosomal dominant pattern, though X-linked and autosomal recessive forms also occur.^12^ Mutations often involve genes encoding cytoskeletal, Z-disk, or sarcomeric proteins (e.g., actin, troponin T, beta-myosin heavy chain, myosin-binding protein C, SCN5A), with sarcomeric variants implicated in 10–20% of inherited pediatric DCM.^13^

Secondary causes of DCM are conditions in which another problem or factor affects the heart muscle leading to ventricular dilation and dysfunction. Neuromuscular disorders represent a significant secondary cause, contributing substantially to morbidity and mortality in affected individuals.^14^ These include Duchenne and Becker muscular dystrophies, limb-girdle muscular dystrophies, and myofibrillar myopathies, exhibiting diverse inheritance patterns. Laminopathies, resulting from mutations in the LMNA gene, can cause DCM often associated with peripheral neuropathy, skeletal muscle disorders, or progeria. Emery-Dreifuss muscular dystrophy is a notable example characterized by skeletal muscle weakness, DCM, and a high prevalence of cardiac conduction abnormalities (e.g., sinus bradycardia, atrioventricular block, atrial fibrillation, ventricular tachycardia), with arrhythmias developing in approximately one-third by age 20 and a significant risk of sudden death.^15^

Additional etiologies for secondary DCM include infective myocarditis, metabolic or mitochondrial disease, infiltrative processes such as amyloidosis, inflammatory processes such as sarcoidosis, nutritional deficiencies, endocrinopathies including thyroid disease, and toxic exposures (e.g., iron overload, lead, cobalt, arsenic, radiation therapy, cardiotoxic chemotherapy including anthracyclines). Mitochondrial diseases, causing impaired cellular energy metabolism, affect the heart in up to 40% of cases, frequently manifesting as a DCM phenotype.^16^

Due to the potential for reversible etiologies of secondary DCM, initial evaluation of a patient with ventricular dilation and dysfunction should include assessment for many of these secondary causes. At our center, initial laboratory screening for new diagnosis includes complete blood count with differential, comprehensive metabolic panel, troponin, creatine kinase, thyroid panel, plasma amino acids, urine organic acids, carnitine and acylcarnitine.

### Hypertrophic cardiomyopathy (HCM)

HCM is characterized by left ventricular hypertrophy in the absence of abnormal loading conditions sufficient to explain the degree of wall thickening.^17^ It must be distinguished from physiological hypertrophy seen in athletes and pathological hypertrophy secondary to conditions like systemic hypertension or aortic stenosis. The primary diagnostic criterion is increased left ventricular wall thickness, typically measured in diastole. While a maximum wall thickness ≥15 mm is often used in adults, pediatric diagnosis relies on Z-scores, with values >2 often considered significant.^7^ The anterior septum is commonly involved, but the pattern and extent of hypertrophy can be highly variable. Systolic function is typically normal or hyperdynamic, although a normal ejection fraction may mask subtle dysfunction in the context of a small, hypercontractile cavity.

HCM pathophysiology often involves progressive hypertrophy, sometimes with dynamic left ventricular outflow tract (LVOT) obstruction. A myocardial oxygen supply/demand mismatch can cause microvascular ischemia, contributing to angina and cardiomyocyte dysfunction or necrosis. This can trigger ventricular arrhythmias and activate pathological remodeling, potentially leading to fibrosis and, in some cases, progression to a dilated phenotype with systolic impairment. Risk stratification for sudden cardiac death is crucial, and implantable cardioverter-defibrillators are indicated based on established risk factors, including the magnitude of hypertrophy, documented ventricular arrhythmias, family history of sudden cardiac death, presence of an apical aneurysm, systolic dysfunction, or certain high-risk genetic mutations.^17^

HCM etiologies can be categorized as primary (often genetic) or secondary (often related to systemic or metabolic disorders). Primary HCM most commonly results from pathogenic variants in genes encoding sarcomeric protein such as MYH7, MYBPC3, TNNT2, TNNI3, TPM1, ACTC1, MYL2, and MYL3.^18^ Inheritance is typically autosomal dominant, exhibiting variable penetrance and expressivity. Despite comprehensive genetic testing, a pathogenic variant is identified in only 50–60% of individuals with clinical HCM. Repeat testing over time may be beneficial as understanding of pathogenic variants expands.

Secondary causes of HCM encompass a range of systemic conditions. Infiltrative and storage diseases are important considerations. Glycogen Storage Disease Type II (Pompe Disease), an autosomal recessive disorder, frequently causes severe HCM, particularly in its infantile form.^19^ Danon Disease, an X-linked dominant condition involving LAMP2 mutations, leads to vacuolar CMP, myopathy, and often Wolff-Parkinson-White syndrome, with a more aggressive course typically seen in males.^20^ PRKAG2 CMP results from mutations affecting AMP-activated protein kinase, causing glycogen accumulation, HCM, and conduction disease. Various lysosomal storage diseases can also manifest with HCM. Mucopolysaccharidoses, such as Hurler syndrome (MPS I) and Hunter syndrome (MPS II), often present with HCM alongside characteristic systemic features and valve abnormalities.^21^ Anderson-Fabry disease, an X-linked recessive sphingolipidosis, typically causes concentric HCM later in life, along with neuropathy and renal disease.^22^

RASopathies represent another significant category of secondary HCM. Noonan syndrome, an autosomal dominant disorder with distinct dysmorphic features, is associated with HCM in 20–30% of cases, often carrying a higher risk of LVOT obstruction and poorer prognosis than non-syndromic HCM.^23^, ^24^

Furthermore, metabolic and mitochondrial disorders can underlie HCM. These include certain fatty acid oxidation defects, like long chain 3-hydroxyacyl-CoA dehydrogenase deficiency, and various primary mitochondrial diseases. Friedreich ataxia, an autosomal recessive neurodegenerative disorder, is frequently associated with HCM often progressing to a DCM phenotype. Finally, endocrine disorders such as hyperinsulinism, particularly in infants of diabetic mothers, and acromegaly can also lead to secondary ventricular hypertrophy.

The prognosis for pediatric HCM varies significantly based on the underlying etiology and age at presentation. Presentation before one year of age generally carries a much worse prognosis. Five-year survival free from death or transplant can range from as low as 42% for HCM related to inborn errors of metabolism to over 94% for those presenting after infancy with other etiologies.^25^ Nevertheless, emerging therapies such as MEK and mTOR inhibitors for RASopathy-associated HCM, small molecules such as cardiac myosin inhibitors for HCM with LVOT obstruction, and RNA-editing tools for in vivo CMP phenotype alteration are actively researched with the potential to reshape the outcomes of this challenging patient population.

### Restrictive cardiomyopathy (RCM)

RCM is a rare but severe form, accounting for approximately 4–5% of pediatric CMP.^26^ It is defined by impaired ventricular compliance and diastolic dysfunction, typically with preserved or near-normal systolic function and ventricular dimensions, leading to progressive biatrial enlargement and elevated filling pressures.^6^ The underlying pathology can involve myocardial fibrosis, infiltrative processes (e.g., amyloidosis, iron overload), or intracellular deposition. Clinical manifestations are broad, including exercise intolerance, respiratory distress, and syncope as manifestations of pulmonary hypertension and inadequate cardiac output, arrhythmias, and sudden cardiac death.^27^ RCM carries the worst prognosis among pediatric CMPs, with approximately 50% mortality or transplantation within 2 years of diagnosis, rising to 80% by 10 years.^26^ Pathogenic variants, often in sarcomeric genes such as MYBPC3 and MYH7, are identified in a significant proportion of cases. Management is challenging, with minimal medical management strategies as patients are often preload-dependent and prone to rapid decompensation, and heart transplantation remains the definitive therapy for end-stage disease.

### Left ventricular noncompaction (LVNC)

LVNC is characterized by a prominent excessive ventricular trabeculations and deep intertrabecular recesses.^28^ As the precise pathomechanism remains unknown, contemporary evidence is pointing away from the hypothesized intrauterine arrest of myocardial development and noncompaction. Instead, excessive ventricular trabeculation likely represents a secondary adaption to a primary genetic or hemodynamic etiology. Officially recognized as a distinct CMP relatively recently, LVNC often coexists with CHDs or overlap with other CMP phenotypes.^29^ Clinical presentations range widely and include arrhythmias, thromboembolism originating from the recesses, and varying degrees of systolic dysfunction leading to heart failure. Pathogenic genetic variants, frequently involving sarcomeric or mitochondrial genes, are identified in roughly half of affected individuals.^28^ The precise LVNC phenotype determines outcomes, with DCM features often portending worse outcomes and higher rates of transplant listing.^8^ Management focuses on degree of CMP, ranging from minimal to no medical management compared to those with ventricular dilation or dysfunction managed with conventional heart failure therapies. Given the increased trabeculations, there is an increased concern for thrombosis, and anticoagulation may be considered in selected patients. LVNC is associated with increased rates of arrythmias including ventricular tachycardia, and as such rhythm monitoring and anti-arrhythmic medications should be considered in these patients.^29^ Transplantation in cases of refractory systolic dysfunction or life-threatening arrhythmias.

### Arrhythmogenic cardiomyopathy (AC)

AC (historically termed arrhythmogenic right ventricular cardiomyopathy), is characterized by progressive fibrofatty replacement of the ventricular myocardium. While classically right-dominant, it can also manifest as a left-dominant or biventricular disease.^30^ AC is a recognized cause of sudden cardiac death, especially in young individuals and athletes.^31^ Many familial cases are linked to mutations in genes encoding desmosomal proteins, such as DSP, DSG2, and PKP2.^32^ The clinical hallmarks are ventricular arrhythmias, syncope, or sudden death, with progressive myocardial involvement eventually leading to heart failure. Diagnosis is established from family history, arrhythmias, cardiac imaging (particularly CMR), endomyocardial biopsy, and genetic testing. Heart transplantation is reserved for patients with end-stage heart failure or medically refractory, life-threatening ventricular arrhythmias.

### Metabolic and specific genetic etiologies

A specific metabolic or syndromic cause is identified in over one-third of pediatric CMPs with a known etiology.^7^ Many of these conditions are detectable through newborn screening programs. Primary carnitine deficiency can present as DCM or HCM and is treatable with oral carnitine supplementation if diagnosed early.^33^ Fatty acid oxidation defects, such as very-long-chain acyl-CoA dehydrogenase deficiency, often manifest as DCM or HCM and may progress rapidly.^10^ Glycogen storage diseases typically cause an initial hypertrophic pattern that can later evolve into DCM. Lysosomal storage diseases include conditions like Gaucher disease, Fabry disease, and various mucopolysaccharidoses. These disorders can lead to restrictive, hypertrophic, or dilated phenotypes due to infiltration and storage within cardiac tissues.^21^, ^22^ Additionally, Barth syndrome, an X-linked disorder, characteristically presents with LVNC, neutropenia, and growth abnormalities. Recognizing these specific diagnoses is crucial. Targeted therapies, such as enzyme replacement, specific diets, or supplementation, may be available for some conditions. An accurate diagnosis significantly impacts clinical management, prognosis, and genetic counseling. It also critically informs decisions regarding the timing or necessity of transplant listing.

## Transplantation and cardiomyopathy

### Heart transplant evaluation

Heart transplantation is considered for children with CMP when medical and surgical therapies are insufficient to maintain adequate cardiac output or when recovery is deemed unlikely (Table 1). Indications for heart transplantation with DCM include Stage D heart failure associated with systemic ventricular dysfunction and with Stage C heart failure with systemic ventricular dysfunction associated with growth failure, severe limitation in physical activity, or life-threatening arrhythmias.^34^ Special consideration should be given for patient selection in the setting of an elevated pulmonary vascular resistance (PVR) since transplantation in the setting of an elevated non-reactive PVR would increase the risk of right ventricular dysfunction of an otherwise unconditioned donor heart.^35^

**Table 1:** Heart Transplant Evaluation and Listing Indications Among Pediatric CMP Patients

CMP	Transplant evaluation/listing indications
DCM	• Stage D heart failure with systemic ventricular dysfunction. • Stage C heart failure with severe limitation in physical activity, or with significant growth failure from heart disease, or with near-sudden death or recalcitrant life-threatening arrhythmias. • UNOS/OPTN Pediatric Status 1A exception pathway for very small children: <5 kg (inpatient) on ≥1 high-dose *or* ≥2 continuous IV inotropes; <10 kg requires additional evidence of poor systemic perfusion; larger children if contraindications to MCS.
HCM	• Stage D heart failure (burned-out HCM) with systemic ventricular dysfunction (LVEF ≤50% is associated with poor prognosis). • Refractory heart failure symptoms or progressive limitation despite maximal therapy; younger HCM patients may progress rapidly—refer early. • High-risk events (cardiac syncope, aborted sudden death, or recalcitrant recurrent/prolonged hemodynamically significant arrhythmias) should prompt urgent evaluation and may support a Pediatric Status 1A exception when inpatient. • Infants (<1 year at initial listing) with HCM qualify automatically for Pediatric Status 1B under OPTN policy; early listing supported by highest waitlist mortality in HCM patients <1 year and high-urgency status.
RCM	• Symptomatic RCM has worse transplant-free survival; presence of *any* cardiac symptoms at diagnosis should trigger transplant evaluation/listing. • Stage C RCM with reactive pulmonary hypertension is a Class I indication; Stage C with risk of evolving fixed PVR is Class IIa—evaluate and list before PVR becomes irreversible. • Candidacy favored when PVR <6 WU m² during vasodilator testing; rising or >6 WU m² supports higher urgency (Status 1A exception) given rising risk for post-transplant right ventricular failure. • UNOS/OPTN Pediatric Status 1A exception considerations for RCM inpatients include syncope, resuscitated sudden death, recurrent hemodynamically significant arrhythmias, and elevated PVR >6 WU m².
General	• Elevated PVR (especially if irreversible) is a *relative to absolute* contraindication to isolated heart transplant; PVR >6 WU m² and/or transpulmonary gradient >15 mm Hg because of high risk of donor right ventricular failure. • Serial cardiac catheterization (including vasoreactivity testing) is recommended in pediatric transplant evaluation to track PVR—particularly in progressive diseases (RCM or HCM) where rising PVR may transition from reversible to prohibitive. • Comprehensive evaluation must address extra-cardiac comorbidities (genetic/metabolic syndromes, end-organ dysfunction), active or recent malignancy, active infection, poor psychosocial support, and other relative/absolute contraindications; decisions made by a multidisciplinary transplant team.

CMP, cardiomyopathy; DCM, dilated cardiomyopathy; HCM, hypertrophic cardiomyopathy; LVEF, left ventricular ejection fraction; MCS, mechanical circulatory support; OPTN, organ procurement and transplantation network; PVR, pulmonary vascular resistance; RCM, restrictive cardiomyopathy; UNOS, united network for organ sharing.

Heart transplantation is indicated for patients with RCM that have pulmonary hypertension responsive to vasoreactivity testing or who are symptomatic with a PVR <6 wood units/m^2^.^36^ Forty percent of pediatric patients diagnosed with RCM have elevated PVR and the extent as well as reversibility determines timing of transplant referral and/or need for VAD.^37^ Interval cardiac catheterizations to determine PVR must be undertaken in asymptomatic patients to allow for timely referral for transplantation. Irreversibly elevated PVR would preclude patients with CMP from heart transplantation.

Evaluation of other comorbidities is important during transplant evaluation. This includes associated genetic or metabolic syndromes with extra-cardiac manifestations, active or ongoing malignancy, and active infection. Patients with end stage heart failure due to advanced neuromuscular disease are typically not considered for heart transplantation given concerns for associated comorbidities, specifically respiratory muscle weakness requiring positive pressure support and severely limited mobility. Nevertheless, transplantation has been successfully reported in patients with Duchenne and Becker’s muscular dystrophy with well-managed respiratory status on non-invasive ventilation.^38^

Risk of waitlist mortality should also be considered when determining the need for advanced cardiac therapies during transplant evaluation. Overall waitlist mortality for pediatric CMP patients is relatively low at approximately 11% for DCM. Mortality risk remains higher for those requiring mechanical ventilation, extracorporeal membrane oxygenation (ECMO), or experiencing active arrhythmias.^4^ Heart failure is the most frequent cause of death while awaiting transplant, followed by cerebrovascular events and multi-organ failure.^4^ Stroke represents the most common cause of waitlist mortality specifically among VAD-supported children.^39^ Encouragingly, waitlist mortality has shown improvement over time for DCM and HCM, though similar trends have not been observed for RCM.^40^ The average time spent on the waitlist for pediatric DCM patients is approximately 9.6 months.^4^ The increasing use of VADs have improved waitlist outcomes especially in the population of pediatric patients with CMP.^41^ Utilization of donor circulatory death hearts in the pediatric population is an emerging practice with likely major implications for waitlist times and mortality.

### Transplant listing status

Pediatric patients with DCM on inotropic support are eligible for united network for organ sharing Pediatric Status 1B listing and Status 1A if requiring mechanical circulatory support. Pediatric patients with DCM are eligible for Status 1A by exception for patients weighing less than 10 kg on continuous inotropic infusion with at least one high-dose inotrope or 2 lower dose intravenous inotropes. Pediatric patients with RCM are specifically eligible for Status 1A by exception if the patients are admitted inpatient with syncopal episodes, had an episode of sudden death or recurrent prolonged runs of hemodynamically significant arrhythmias refractory to medical therapy and/or evidence of elevated PVR (>6 WU*m^2^).^42^ These criteria reflect the 2016 allocation changes, which produced a 13-fold rise in Status 1A exceptions for DCM and only a 2- to 3-fold rise for HCM and RCM patients. Because HCM and RCM patients are less well-supported on VADs, this smaller increase in priority translated into higher wait-list mortality for these groups, whereas DCM patients benefited from both more frequent exceptions and reliable VAD bridging.^43^, ^44^

### Considerations for mechanical circulatory support

Optimal medical management of patients with CMP often is not sufficient to allow for stabilization and bridge to transplantation. VADs have significantly improved outcomes for children awaiting transplant, proving superior to ECMO as a long-term bridging strategy.^39^, ^45^

Indications for use of durable mechanical circulatory support in this population include the following: respiratory decompensation requiring escalation is support, end organ dysfunction in the form of liver and/or renal injury, feeding intolerance and need for nutritional rehabilitation.^46^ Patients with end organ injury require timely implantation of VAD support to ensure reversal of dysfunction and allow for improved outcomes.^47^, ^48^ Patients with CMP with elevated PVR require VAD as a bridge to candidacy to allow for left sided decompression.

Approximately 15% of children with DCM listed for transplant receive VAD support^4^ with similar long-term post-transplant survival to DCM recipients without VADs.^4^ Similarly, while VAD use is less common in children with RCM and HCM (approximately 3% compared to 23% for DCM), it does not negatively impact waitlist survival in this population.^40^

### Transplant outcomes for pediatric cardiomyopathy

**Figure 1:**
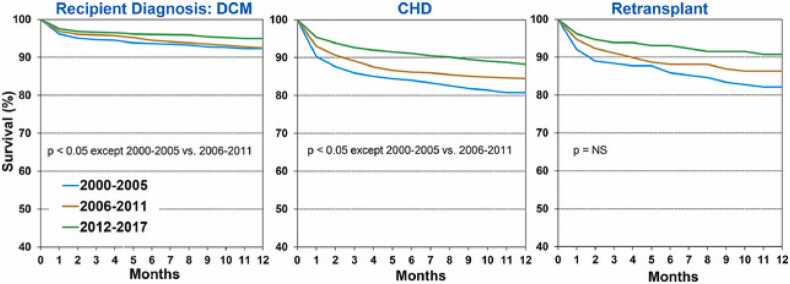
From Singh et al., 2021, ISHLT Registry 24th Pediatric Heart Transplantation Report, https://doi.org/10.1016/j.healun.2021.07.022. ISHLT, International Society for Heart and Lung Transplantation.

Children transplanted for CMP experience excellent outcomes. Recipients with DCM tend to have superior 1- and 5-year survival compared to those transplanted for complex CHD (Figure 1). The reasons are multifactorial including but not limited to fewer prior sternotomies and less complex surgical anatomy.^49^ Post-transplant survival for CMP patients has steadily improved since the early 1990s.^4^, ^40^ Contemporary data indicate that 5-year survival for RCM and HCM recipients improved from less than 80% in the 1990s to over 90% in the 2010s.^40^, ^50^ Long-term survival for pediatric CMP recipients discharged after transplant is favorable, with reports of up to 72% survival at 15 years post-transplant, showing minimal differences between dilated and restrictive subtypes in some cohorts.^4^, ^49^

## Conclusions

Pediatric CMP is a heterogeneous disease with highly variable clinical phenotypes. Heart transplantation is an excellent therapeutic strategy for patients with progressive CMP refractory to conservative management. DCM is the most common indication for transplantation with HCM and RCM increasing in frequency over the past 2 decades. Waitlist mortality has remained low with VAD increasingly emerging as a viable bridge to transplantation.

## Declaration of Competing Interest

The authors declare that they have no known competing financial interests or personal relationships that could have appeared to influence the work reported in this paper.
